# Gender effect on clinical features of achalasia: a prospective study

**DOI:** 10.1186/1471-230X-6-12

**Published:** 2006-04-01

**Authors:** Javad Mikaeli, Farnoosh Farrokhi, Faraz Bishehsari, Mahboobeh Mahdavinia, Reza Malekzadeh

**Affiliations:** 1 Achalasia Research Unit, Digestive Disease Research Center, Tehran University of Medical Sciences, Shariati Hospital, Tehran 14114, Iran

## Abstract

**Background:**

Achalasia is a well-characterized esophageal motor disorder but the rarity of the disease limits performing large studies on its demographic and clinical features.

**Methods:**

Prospectively, 213 achalasia patients (110 men and 103 women) were enrolled in the study. The diagnosis established by clinical, radiographic, and endoscopic as well as manometry criteria. All patients underwent a pre-designed clinical evaluation before and within 6 months after the treatment.

**Results:**

Solid dysphagia was the most common clinical symptom in men and women. Chest pain was the only symptom which was significantly different between two groups and was more complained by women than men (70.9% vs. 54.5% P value= 0.03). Although the occurrence of chest pain significantly reduced after treatment in both groups (P < 0.001), it was still higher among women (32% vs. 20.9% P value= 0.04). In both sexes, chest pain did not relate to the symptom duration, LES pressure and type of treatment patients received. Also no significant relation was found between chest pain and other symptoms expressed by men and women before and after treatment. Chest pain was less frequently reported by patients over 56 yrs of age in comparison to those less than 56 yrs (p < 0.05).

**Conclusion:**

It seems that chest pain is the distinct symptom of achalasia which is affected by sex as well as age and does not relate to the duration of illness, LESP and the type of treatment achalasia patients receive.

## Background

Achalasia is a primary esophageal motor disorder of unknown etiology characterized by absent esophageal peristalsis and abnormal lower esophageal sphincter (LES) relaxation [1-3]. The disease can occur at any age but it is usually diagnosed in patients who are between 25 and 60 years. The motor abnormalities found in this disease compromise the normal esophageal emptying and result in several clinical symptoms including dysphagia as the most common symptom, postprandial and/or nocturnal regurgitation, weight loss, chest pain and cough [4-7].

Men and women are affected with equal frequencies [8], but data regarding the characteristics differences between males and females in achalasia is spars.

Although studies on esophageal motility in normal individuals have shown some differences between males and females [9], this was not widely studied among achalasia patients. Demographic studies in achalasia have been hampered by low incidence and rarity of the disease, which limits the number of cases to be studied and therefore weakens the probability to find significant differences in subgroups of achalasia patients.

Our center as the major referral center for achalasia in Iran provides us with the opportunity to include relatively large number of patients during these years. In this study, we aimed to evaluate the differences of the clinical features between men and women before and after treatment, and assess whether the dissimilarities are related to the clinical course, demographic features, or manometric characteristics, and determine how treatment affects the discrepancies.

## Methods

### Patients

Over a period of ten years from July 1994 to December 2004 all symptomatic patients with idiopathic achalasia referred to the Shariati Hospital of Tehran University of Medical Sciences were consecutively enrolled in the study. The diagnosis of achalasia was established by clinical (dysphagia, regurgitation, chest pain), radiographic (bird's beak appearance of the lower esophageal sphincter, decreased esophageal peristalsis, delayed esophageal emptying) and endoscopic as well as manometric criteria (aperistalsis of the esophageal body, increased lower esophageal sphincter pressure and incomplete relaxation (<50%) on swallowing).

The study protocol was reviewed and approved by the ethic committee of digestive disease research center of Tehran University of Medical Sciences and informed consent was obtained from all patients.

### Clinical evaluations

A structured interview evaluating demographic as well as clinical features was conducted at the time of their first visit and within 6 months following their initial treatment (before the second treatment following relapse), and the standard questionnaire was filled for each of the patients. Evaluation of their clinical features was done on the bases of five clinical symptoms; solid and liquid dysphagia, active and passive regurgitation and chest pain. The frequency of each of these symptoms was scored on a scale of 0–3 to obtain the total symptomatic score, as described elsewhere [10,11] (table 1). The highest obtainable score was 15.

Additionally the history of other symptoms such as nocturnal cough, weight loss as well as any evidence of the previous treatment was taken.

The patients underwent botulinum toxin injection or pneumatic dilation as described bellow.

Subsequently the total symptomatic score was compared among men and women patients before and within 6 months after the treatment.

### Esophageal manometry

Manometric assessment was added to achalasia patient's evaluation from 1997 in our center. After an overnight fast, manometry was carried out by a standard eight channel low compliance catheter (Synecticw, Synectics, Stockholm, Sweden) perfused with hydraulic pump. Lower esophageal sphincter pressure (LESP) was studied by rapid pull through (RPT) technique in deep expiration. The average of five RPT analyses was recorded as resting LESP. An average of 10 water swallows was used to assess body contraction waves and LES relaxation.

### Botulinm toxin injection

Botulinum toxin injection (BT) was offered as the therapeutic choice in achalasia patients with tortuous megaesophagus, epiphrenic diverticula, previous cardiomyotomy, age > 60 yrs with cardiopulmonary or comorbid diseases. Four-hundred units of Dysport (Ipsen, UK) equal to 160 units of Botox [12] was injected following an overnight fast, using 100 units in each quadrant at the level of LES under videoendoscopic guide.

### Pneumatic dilatation

After a clear liquid diet for 12 h and an overnight fast, pneumatic dilatations (PD) were performed using 35 or 30 mm Rigiflex balloon (The technique changed from using 35 mm balloon diameter to 30 mm diameter Since 1997) under a conscious sedation with diazepam and meperidin (5–10 and 25–50 mg I.V respectively). Following a complete upper gastrointestinal endoscopy, balloon dilators were passed over a guide wire and were positioned such that LES places at the midpoint of the balloon under videoendoscopic guide. Rigiflex balloons were gradually inflated up to 10 psi in 30s and maintained for another 60 s. After emptying and pulling out the balloons, patients were endoscoped again to assess the LES opening (relaxation) and any evidence of bleeding or perforation and were discharged after a 6 h observation if remained asymptomatic.

### Statistical analysis

Continuous variables were summarized as mean ± standard deviation. Qualitative variables were summarized as a percentage of the group total. Comparing numerical variables with assumed equal variances and different variances tested by t student and mann-withney respectively.

A p-value of less than 0.05 was considered significant.

## Results

213 patients including 110 (51.6%) men and 103 (48.4%) women were enrolled in the study. No significant differences were found in the characteristics of men and women before their initial treatment (table 2).

The frequencies of the clinical symptoms were compared between men and women before and within 6 months of treatment.

### Before treatment

Solid dysphagia was noted as the first clinical symptom in the disease course in 81% of men and 76.4% of women. No significant difference was found in the clinical features of achalasia between two groups except for the chest pain which was more common in women than in men (70.9% vs. 54.5% *P *= 0.03) (table 3).

LESP was measured in 103 patients (50 women and 53 men), Mean LESP was 53.7 ± 17.4 and 58.6 ± 19.5 mmHg in men and women respectively (*P *= 0.1). The LESP did not differ significantly between patients with and without chest pain, neither in men nor in women (*P *= 0.6 & 0.8 respectively).

Out of 110 men and 103 women, 47.3% vs. 54.4% had received no previous treatment, 3.6% vs. 10.6% had undergone surgical myotomy, 47.3% vs. 30.2% had been recommended to use medications such as nitrates or calcium channel blockers, 0.9% vs. 1.9% and 0.9% vs. 2.9% had undergone previous PD and BT respectively.

The frequency of chest pain experienced by men and women had no significant relationship with the type of pretreatment therapy they had received (*P *values > 0.05).

Occurrence of chest pain did not relate to the symptomatic duration before treatment in both sexes; mean symptom duration in women with (n = 73) and without chest pain (n = 30) was 4.5 ± 3.6 and 5.5 ± 6.2 yrs respectively (*P *= 0.3), while 4.5 ± 4.7 and 4.7 ± 6.4 yrs in 60 men with and 50 men without chest pain (*P *= 0.8).

No significant relation was found between chest pain and other symptoms such as solid dysphagia, liquid dysphagia, active and passive regurgitation (*P *> 0.05 in all cases).

Chest pain was less frequently reported by patients over 56 yrs of age in comparison to those less than 56 yrs (42.8% vs. 65.5% *P *= 0.02).

### After treatment

In total, 171 (80.5%) patients underwent pneumatic dilatation and 42 (19.5%) underwent botulinum toxin injection; 62.4% and18.8 % of patients underwent one and two sessions of PD, and 12.2% and 5.6% of the cases received one and two BT injections respectively.

Symptoms of the patients were evaluated again at 6 months after treatment, as depicted in table 4. Frequency of chest pain after treatment did not relate to the type of therapy that achalasia patients received (*P *= 0.4). There was no significant relation between chest pain and other symptoms such as solid dysphagia, liquid dysphagia, active and passive regurgitation (all *P *values > 0.05).

The occurrence of chest pain significantly reduced after treatment in both sexes (*P *= 0.0001) but it was still higher among women (32% vs. 20.9% *P *= 0.04).

During 6 months after treatment, the occurrence of chest pain in patients over 56 was significantly lower in comparison to younger group; 7.1% vs. 29.2% *P *= 0.01

## Discussion

We evaluated the clinical features of achalasia in relation to the demographic aspects of the affected individuals, studying the consecutively enrolled achalasia patients in our center. Potential epidemiological limitations of studies on rare diseases as achalasia include inadequate sample size and the consequent selective presentation of results due to multiple comparisons. Regarding to these concerns, we avoided multilayer comparisons and tried to limit the number of comparisons in order to be supported by our sample size. We have tried to evaluate the clinical profile of our patients in detail, but more extensive questionnaire could be more informative in term of finding differences between men and women.

It has been suggested that gender and age affect the clinical presentation of achalasia [13-15]. A study by d' Alteroche et al. showed that chest pain was 1.7 times more frequent in women than in men, but no explanation was suggested by the authors [14].

In the present study, we found chest pain is the only symptom that differs between men and women; it is more common in women before or after treatment. The occurrence of chest pain did not relate to the LESP in both sexes.

Some previous studies have tried to figure out the underlying causes of chest pain in achalasia patients. Eckardt et al evaluated achalasia patients with and without complaining chest pain and found that the occurrence of this symptom was unrelated to LESP. In addition, They showed that repetitive esophageal contractions were more common in patients experiencing chest pain, although this finding barely reached the specific cut off [16]. Another study found that 38% of patients with reappearance of peristalsis waves in manometry performed 1 year after dilation experienced the symptom of chest pain, while only 6% of those with esophageal aperistalsis complained about this symptom. In addition, post dilation peristalsis was strongly correlated to higher contraction waves amplitude before treatment [6].

Results of Dantas et al. study on normal subjects could help to explain the observed difference in frequency of chest pain between men and women in our series. They performed manometry on 20 men and 20 women with the same age distribution. There was no difference in LESP between men and women, but a higher duration of contractions 5 cm above the LES was observed in women (women: 4.5 ± 0.3s men: 3.7 ± 0.2s p < 0.05). On the other hand no gender difference in esophageal functional anatomy or innervations has been described [9].

All these data suggest that higher occurrence of chest pain among women may be interrelated to higher amounts or amplitude of contractions in this group.

In addition, women are more likely to experience recurrent pains or report more intense and longer duration pain comparing to men [17,18]. This might be due to some biological and psychological differences between these two groups which can lead to lower pain tolerance and threshold in women [18]. Thus the different pain expression in women could also play a role in the difference seen in the chest pain between two genders in achalasia.

We found that chest pain is conversely related to the age and is less frequent in elderly.

Previous studies also pointed that chest pain decreases significantly with ageing [14,16].

Clouse and Simmons reported that not only chest pain is less frequent in elderly subjects but also the pain is less sever in this age group[13,19]. It has been shown that there is no difference in LESP and other manometric features in older comparing younger achalasia patients except the lower LES residual pressure in elderly [13,20]. The lower LES residual pressure along with lesser visceral perception in elderly were considered to explain lesser expression of chest pain in the older achalasia patients [16].

In agreement with previous studies, our results showed that the occurrence and intensity of chest pain was unrelated to the intensity of other esophageal symptoms [16]. This was the case in both sexes, either before or after treatment.

There was no significant relationship between symptom duration and chest pain in our study. Rakita et al. and Alteroche et al. also found that duration of symptoms had little impact on premyotomy symptom frequency [14,15]. However, results of the other study showed that patients with chest pain had a shorter duration of symptoms before the diagnosis of achalasia. The authors suggested that more pronounced disease in patients with chest pain could call for sooner investigation to receive a diagnosis at an earlier stage [16].

Some studies have indicated that a successful treatment improves chest pain but rarely makes this symptom abolish [1,16,21,22]. We found that occurrence of chest pain decreased after therapeutic session in both sexes, but remained still higher among women. The occurrence of other symptoms was not different between men and women after therapy. This shows that not only chest pain is more frequent in women but also responds less favorable to the current treatments. It has been previously suggested that these patients could also benefit from accompanying behavioral and emotional treatments to reduce their pain [22,23]. Although around 70% of patients in our study reported some extent of solid dysphagia after therapy, the total dysphagia score has significantly decreased after treatment and only in 18% of patients the dysphagia score was greater than 1.

Many efforts have been done in treatment of achalasia during the past decade, but the affected patients still suffer from chest pain even after otherwise successful treatment. Timed barium swallow as an objective means of assessing esophageal emptying would show whether the continued chest pain in achalasia patients after treatment is associated with unfavorable esophageal emptying. Studies to clarify the underlying mechanism of this symptom would help us to perform more efficient managements for achalasia.

## Conclusion

It seems that chest pain is the only symptom of achalasia which is affected by sex as well as age. The duration of illness, lower esophageal sphincter pressure and the type of therapeutic procedure do not relate to the occurrence of chest pain in achalasia and can not explain its higher occurrence in women. The relation between chest pain and sex, observed in this study needs to be explored in detail to understand the physiologic- based content of this symptom in achalasia.

## Competing interests

The author(s) declare that they have no competing interests.

## Authors' contributions

JM visited the patients and performed endoscopies and therapeutic procedures.

FF conducted the study, interviewed the patients, analyzed the data, and drafted the manuscript.

FB assisted in conducting the study and interviewing the patients and revised the manuscript.

MM assisted in interviewing the patients and helped in revising the manuscript.

RM supervised the study scientifically, has been involved in preparing the manuscript and has given final approval of the version to be published.

All authors read and approved the final manuscript.

## Pre-publication history

The pre-publication history for this paper can be accessed here:



## Figures and Tables

**Table 1:** Scoring system for evaluation of clinical symptoms

Score	0	1	2	3
Symptoms				
Dysphagia to solids	none	weekly	Daily	each meal
Dysphagia to liquids	none	weekly	Daily	each meal
Active regurgitation	none	weekly	Daily	each meal
Passive regurgitation	none	monthly	Weekly	daily
Chest pain	none	monthly	Weekly	daily

**Table 2:** Comparing the characteristics between men and women before the initial treatment

Variable	Women (n = 103, 48.4%)	Men (n = 110, 51.6%)	P Value
Age (SD) (min-max)	39.3 (14.5) (16–77 y)	37.7 (14.1) (14–75 y)	NS* (0.4)
Mean symptom duration (min-max) (Years)	4.8 (0.5–28)	4.6 (0.4–30)	NS (0.9)
Mean weight loss (Kg) (SD)	3.2 (4)	3.2 (4.9)	NS (0.7)

NS: Not Significant

**Table 3:** Comparing the presenting symptoms between men and women before therapy

Variable		Women (n = 103, 48.4%)		Men (n = 110, 51.6%)		P Value
Mean pre treatment total score (SD)		9.9 (2.7)		9 (3.1)		0.01
Pre treatment Solid dysphagia		100%		96.4%		NS (0.2)
Pre treatment Liquid dysphagia		98.1%		90.9%		NS (0.5)
Pre treatment active regurgitation		71.8%		73.6%		NS (0.2)
Pre treatment passive regurgitation		77.7%		63.6%		NS (0.2)
Pre treatment chest pain	Daily	12.6%	70.9%	8.2%	54.5%	0.03
	Weekly	44.7%		38.2%		
	Monthly	13.6%		8.1%		
	Never	29.1%		45.5%		
Nocturnal cough		51.5%		40%		NS (0.09)

NS: Not Significant

**Table 4:** Comparing the presenting symptoms between men and women after treatment

Variable		Women (n= 103, 48.4%)		Men (n = 110, 51.6%)		P Value
Mean post treatment total score (SD)		2.4 (2.2)		2.7 (2.7)		NS (0.3)
Post solid dysphagia		68.9%		76.4%		NS (0.1)
Post liquid dysphagia		25.2%		33.6%		NS (0.09)
Post active regurgitation		12.6%		15.5%		NS (0.5)
Post passive regurgitation		7.8%		8.2%		NS (0.9)
Post chest pain	Daily	1%	32%	0%	20.9%	0.04
	Weekly	11.7%		5.4%		
	Monthly	19.3%		15.5%		
	Never	68%		79.1%		

NS: Not Significant
